# Network Theory Inspired Analysis of Time-Resolved Expression Data Reveals Key Players Guiding *P. patens* Stem Cell Development

**DOI:** 10.1371/journal.pone.0060494

**Published:** 2013-04-18

**Authors:** Hauke Busch, Melanie Boerries, Jie Bao, Sebastian T. Hanke, Manuel Hiss, Theodhor Tiko, Stefan A. Rensing

**Affiliations:** 1 ZBSA Center for Biological Systems Analysis, University of Freiburg, Freiburg, Germany; 2 FRISYS Freiburg Initiative for Systems Biology, University of Freiburg, Freiburg, Germany; 3 FRIAS Freiburg Institute for Advanced Studies, University of Freiburg, Freiburg, Germany; 4 Institute of Molecular Medicine and Cell Research, Albert-Ludwigs-University Freiburg, Freiburg, Germany; 5 German Cancer Consortium (DKTK), Freiburg, Germany; 6 German Cancer Research Center (DKFZ), Heidelberg, Germany; 7 Faculty of Biology, University of Freiburg, Freiburg, Germany; 8 Faculty of Biology, University of Marburg, Marburg, Germany; 9 BIOSS Centre for Biological Signalling Studies, University of Freiburg, Freiburg, Germany; University of Pittsburgh, United States of America

## Abstract

Transcription factors (TFs) often trigger developmental decisions, yet, their transcripts are often only moderately regulated and thus not easily detected by conventional statistics on expression data. Here we present a method that allows to determine such genes based on trajectory analysis of time-resolved transcriptome data. As a proof of principle, we have analysed apical stem cells of filamentous moss (*P. patens*) protonemata that develop from leaflets upon their detachment from the plant. By our novel correlation analysis of the post detachment transcriptome kinetics we predict five out of 1,058 TFs to be involved in the signaling leading to the establishment of pluripotency. Among the predicted regulators is the basic helix loop helix TF PpRSL1, which we show to be involved in the establishment of apical stem cells in *P. patens*. Our methodology is expected to aid analysis of key players of developmental decisions in complex plant and animal systems.

## Introduction

Plant cells differentiate from stem cells into specialized tissue cells and back to cope with varying environmental cues. The juvenile form of the moss *Physcomitrella patens* are the filamentous protonemata. Each filament extends by tip growth through unequal division of the apical stem cell [1]. Upon detachment of leaflets (phyllids) from the plant, individual leaflet cells undergo transdifferentiation and eventually become an apical stem cell [2], [3], which in turn generates a new protonemal filament through unequal division. While the coarse timeframe of *P. patens* leaflet cell transdifferentiation has been studied, the dynamic interplay of gene expression and transcription factor (TF) regulation during this decision process remains poorly understood. One viable approach to elucidate the sequential activation of signaling processes involved in cell (de-)differentiation is the use of time-resolved microarray data. Indeed, processes in animal cells that evolve on time scales of hours to days, like differentiation, have been shown to exhibit a strong correlation between transcriptome kinetics, *de novo* protein synthesis and long-term cell behavior [4], [5], [6]. This finding is possibly rooted in the fact that complex regulatory networks are controlled by their slowest evolving subsystems [4], [7]. Here, we assumed the same to be true for plant cell dynamics.

Although transcriptome actions are important, the transcriptome response of a cell provides only an incomplete picture of cellular events. Many fast processes are preferentially regulated on the proteome level. Among those are protein modification or the regulation of TF activity. Therefore, the actual mechanisms leading to the observed transcriptome responses might remain elusive if considering the strongest responding genes alone. This behavior is probably rooted in the topology of the underlying gene and protein regulatory networks. Comparing gene expression in healthy versus diseased specimens, it was found that the change in gene expression as well as expression variability correlates with network connectivity, i.e. the number of interaction partners of the encoded proteins [8], [9]. Moreover, strongly differentially regulated, yet weakly connected genes, show biological functions tightly linked to the phenotype, whereas hub genes tend to have more general functions. De-regulated effectors of diseases like cancer are preferentially such (typically weakly regulated, but strongly connected) network hubs [10].

To capture the coordinated response of a cell, the concept of cellular attractors has been suggested. Similar to complex networks settling on a small number of stable configurations [11], it has been suggested that complex gene regulatory networks possess a finite number of stable expression profiles, called cell attractors. They are thought to coordinate and guide cellular decisions, such as differentiation. While the biological mechanisms that define these attractors remain abstract, they aid in understanding global transcriptome response patterns in animal cells enabling the identification of genes putatively involved in cellular decisions, irrespective of their differential expression strength [12]. In the following we suggest to combine analysis of differentially regulated genes with cell attractor reconstruction to identify weakly regulated key players mediating the dynamic differentiation process.

In this study, we apply these systems theoretic ideas for the first time in plant cells to elucidate the formation of *P. patens* apical stem cells upon leaflet detachment. Using time-resolved microarray expression profiling, we identify key signaling components and mechanisms of an important plant developmental process by means of multidimensional scaling analysis of gene expression time series data in combination with a correlation-based analysis of global transcriptome-response behavior. The approach singles out genes with weak to moderate differential expression that control the developmental progression and its underlying transcriptional control. In this way, we predict and experimentally verify TFs critically involved in (or excluded from) the cellular decision of *P. patens* leaflet cells to undergo transdifferentiation into apical stem cells. Validation of loss of function mutants demonstrates for two examples that the prediction holds true.

## Results and Discussion

### Time-resolved microarray data of detached leaflet transdifferentiation

**Figure 1:**
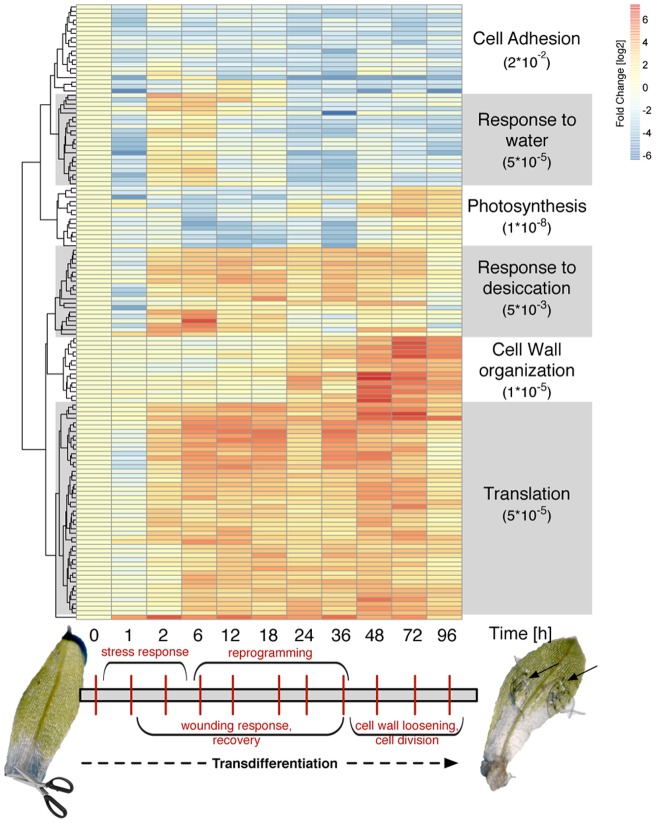
Transcriptome response of *P. patens* leaflets upon detachment. Heatmap of significantly responding genes from leaflet detachment to eventual cell division and apical filamentous growth (0–96 h a.d.). The terms on the right denote the most significant GO biological processes of the six largest clusters together with their respective p-value using conditional hypergeometric testing. The grey bar below depicts the overall sequence of events as derived from the GO analysis. The micrographs show detached leaflets at time point 0 h and 72 h, respectively; black arrows indicate filaments protruding from the leaflet.

To study the time-resolved development of apical stem cells, we detached leaflets of *P. patens* gametophores, isolated RNA 0–96 h after detachment (a.d.), and subjected it to microarray analysis [13]. Previous analyses of detached leaflets that transdifferentiate into apical stem cells showed the importance of the *P. patens* ortholog of the polycomb group protein FERTILIZATION INDEPENDENT ENDOSPERM (FIE) as a marker for newly reprogrammed stem cells [2]. Its appearance 48 h after detachment (a.d.) defines the time window of epigenetic reprogramming between initial signaling and unequal division of the newly formed apical stem cell. Here, we found that the majority of leaflets have developed at least one apical stem cell at 72 h a.d. (Fig. 1), as evidenced by the emergence of tip-growing filaments. Based on this time scale for apical stem cell development, we collected leaflet RNA at 0, 1, 2, 6, 12, 18, 24, 36, 48, 72 and 96 h a.d. Weakly expressed genes and genes with low inter-array variability were filtered out, leaving 17,158 out of 27,715 transcripts for subsequent analysis. The 0 h time point was taken in duplicate to assess reproducibility of the fold expression values of the experiments, since each time point taken effectively represents an independent biological sample. Comparing the fold change of the whole transcriptome with respect to the two microarrays taken at 0 h one finds clearly reproducible results (Fig. S1). In the following we averaged the expression values for each gene at 0 h to calculate the respective fold change for all subsequent time points. The lack of time point replicates, despite for 0 h, represents a potential shortcoming of our design, since no classical statistical approaches can be performed on the data and it might contain undetected outlier samples. However, we believe that the design of the timecourse experiment and the subsequent analysis approach conducted here balances this potential weakness. Any putatively failed experiment time point would show in having a disproportionally large number of outlier genes, which we did not find to be the case. Moreover, our microarray data confirmed the activation and time course of two previously reported markers for transdifferentiation: the expression of *CYCD;1* (Phypa_226408) [3] and the *FIE* transcript, Phypa_61985 [2], being continuously up-regulated over the whole time course, respectively transiently up-regulated between 6–36 h a.d. (Fig. S2A). This demonstrates that although a minority of leaflet cells is strongly expressing *CYCD;1* and *FIE*, the RNA derived from the mixed cell population can be employed to detect expression profiles of such a subset.

**Figure 2:**
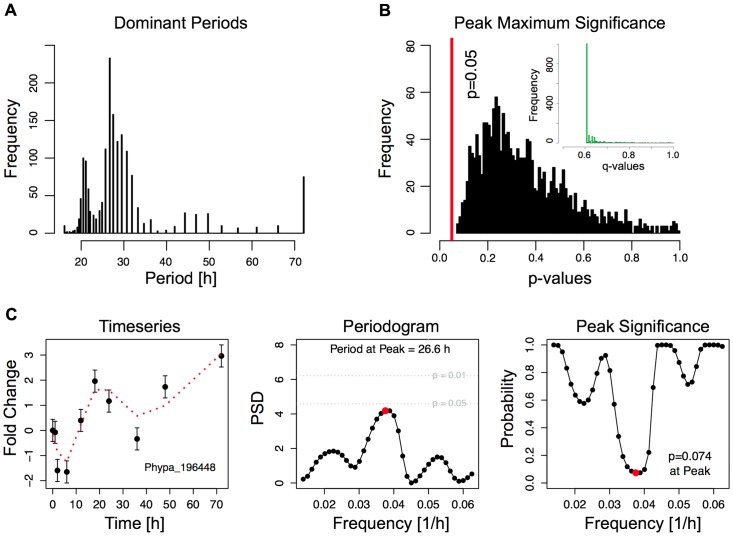
Spectral analysis of gene expression time series via Lomb-Scargle periodograms. **A**, Histogram of dominant oscillation periods in the power spectral densities of the 1,500 most significantly regulated genes as evaluated from the MDS analysis. The dominant periods have been determined from the peak in the Lomb-Scargle periodogram of each gene. **B**, Histogram of p-values denoting the statistical significance of the dominant periods in the Lomb-Scargle periodograms for each gene. None of the genes has a peak significance p<0.05 (marked by the vertical red line). The insert shows the histogram of the FDR-corrected p-values for the set of 1,500 genes. **C**, Example analysis for Phypa_196448, which has the lowest p-value p = 0.074. Left: Phypa_196448 time series and corresponding fit using a 5^th^ order polynomial regression model (red dashed line). Error bars have been estimated from the variance of expression for the 0 h time point that was taken in duplicate. Middle: Normalized power spectral density (PSD) of the Lomb-Scargle periodogram. The red dot marks the dominating frequency of the periodogram. Right: Significance of Lomb-Scargle frequencies. The red dot marks the dominating frequency of the Lomb-Scargle periodogram.

Circadian rhythms can play an influential role in gene expression dynamics of plants [14], [15], [16], [17]. Since we sampled the leaflet response for several days we searched for an apparent 24 h oscillation underlying the global gene expression, to rule out a significant influence of circadian rhythm on the transcriptome response. As the data points are unevenly spaced, a Lomb-Scargle periodogram was calculated for each gene. We tested and rejected the null hypothesis that a gene is periodic by calculating how likely an observed peak in the Lomb-Scargle periodogram occurs by chance [18]. None of the genes were found to have a significant period of 24 h (lowest p-value >0.07 and lowest FDR corrected q-value >0.605, Fig. 2B). Hence, circadian rhythm does not seem to have a dominant effect and the apparent gene regulation is mainly due to the stress signaling and transdifferentiation processes induced by leaflet detachment. We hypothesize that the detachment resets the circadian clock and that periodicity will be established only later, subsequent to the tissue regaining a normal state. Besides the above mentioned analysis, this is also supported by the fact that for several genes previously shown to exhibit periodicity in *P. patens* [17], [19], [20], [21], [22] no circadian or diurnal rhythm could be detected in our data, despite the fact that a long day light regime was employed.

### Gene Ontology bias analyses reveal the chain of events

To assess the biological significance of gene regulation from detachment to eventual cell division and filamentous growth per time point we performed conditional hypergeometric testing for Gene Ontology (GO) bias [23]. Hierarchical clustering of the expression kinetics shows a functional sequence of events for transdifferentiation (Fig. 1). Analysis for the first 6 h a.d. finds genes encoding for dehydrins, desiccation related proteins, late embryogenesis abundant (LEA) proteins, catalases or early response to dehydration (ERD) proteins as enriched (GO terms: response to water, response to desiccation; Fig. S3). This reflects the immediate stress response of the detached leaflets. Up to 24 h, genes associated with energy metabolism and translation are up-regulated, while photosynthesis is down-regulated. In this phase the leaflet cells apparently process the physical wounding and stress signals and make the decision towards pluripotency. At 36 h a.d. regulation of DNA replication is enhanced, marking a turning point by preparing for cell division. After 36 h, energy metabolism is still predominant. Additionally, modification of cell walls starts and photosynthesis restarts after 48 h, suggesting the execution of cell division and formation of the apical stem cells (Fig. 1, S3). Recently, transcriptome analysis of *P. patens* protoplast transdifferentation into tip growing protonemata has been conducted [24]. While there is some resemblance in terms of GO bias at later stages, no overlap of short term data is evident, probably due to the severely different *in vitro* start conditions. While in our study leaflets were detached from precultured gametophores, exerting primarily wounding and drought stress, the protoplastation (enzymatic removal of the cell wall under hypoosmotic conditions) obviously represents quite a different stress scenario. Moreover, the culture media and conditions are different, most notably with regard to the presence of ammonium tartrate in the other study.

### Wounding and osmotic/drought stress trigger the reprogramming

While the importance of D-type cyclin and cyclin-dependent kinase A activation for transdifferentiation has recently been shown [3], the initial trigger for this process upon leaflet detachment is largely unknown. As our functional analysis predicted an involvement of osmotic/drought stress, we checked whether this is an obligatory signal towards pluripotency establishment. We compared detached leaflets in liquid mineral medium (representing a hypo-osmotic condition) and under iso-osmotic conditions. Under hypo-osmotic conditions, 81% of leaflets (n = 26) had formed filaments 120 h a.d., while under iso-osmotic conditions not a single leaflet (n = 25) did. Gametophores grown under standard conditions over several weeks did not exhibit formation of filaments from leaflets. In contrast, when grown under mildly dehydrating conditions over the same time period, protonemal filaments occasionally arose from the gametophore leaflets (Fig. S4). Taken together, the osmotic/drought pathway and the wounding response are both necessary triggers for pluripotency establishment. Such a failsafe situation makes sense to prevent reprogramming of differentiated cells under normal conditions.

### Defining moderately regulated genes following the global transcriptome trend

Due to the long time scale of observation (four days), a strong correlation between transcriptome response and phenotype is expected [4], [6]. Therefore, we utilized a multi-dimensional scaling analysis (MDS) to identify strongly and uniquely responding genes, assuming them to be key to the stress response and cell differentiation. Here, we use MDS to map the matrix of all pairwise Euclidean distances between gene expression time series onto a two-dimensional space. We identified 299 significantly responding genes by fitting a multivariate skew-normal distribution to the resulting two-dimensional MDS distribution (q-value <0.05) (Fig. S2B); such genes provide a marked link to the cellular transdifferentiation phenotype development (Fig. 1). However, GO terms for transcriptional processes, regulation of gene expression, DNA methylation and chromatin organization, all of which are expected to point to the underlying transdifferentiation control, are underrepresented among these (Table S1). Genes important for development, such as TFs, are mostly weak to moderately strong regulated, and thus are problematic to detect by standard analyses. To overcome this problem we hypothesize (i) that plant cell transdifferentiation proceeds along a coordinated change of the whole transcriptome and (ii) that genes putatively coordinating this process follow the global change of the transcriptome. This hypothesis is supported in the light of the cell attractor idea, should apical stem cell development bear similarities to self-organization in complex systems. There, few variables, called order parameters, become unstable upon stimulation and control the transition dynamics of the whole system [4]. Akin to this, only few genes were found to be strongly responding during transdifferentiation (Fig. S2A), correlating well with the cellular phenotype development over time. Turning this argument around, genes reflecting the mechanisms leading to transdifferentiation, like signal transduction cascades, should have comparatively low differential response strength but should follow the global trend of the transcriptome.

**Figure 3:**
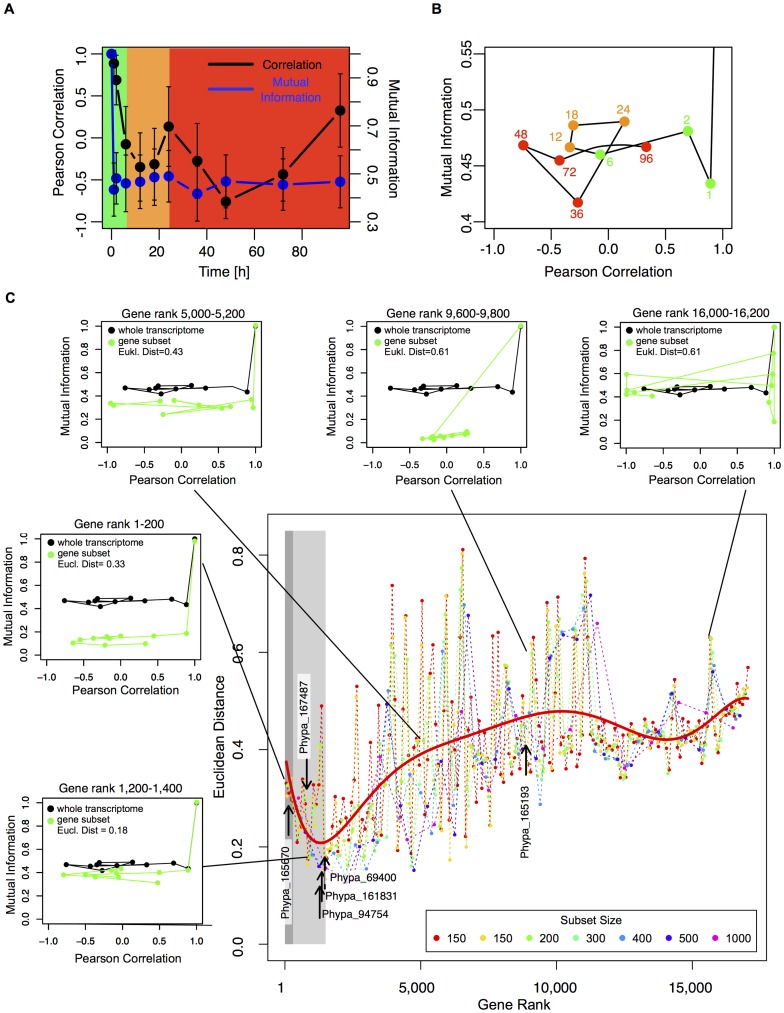
Correlation analysis of whole transcriptome and ranked gene subsets. **A**, Whole transcriptome Pearson correlation (*r_v,_* black curve) and mutual information (*I, blue curve*) for all measured time points with respect to 0 h. The dots and error bars denote the mean and standard deviations from 1,000 random samples using a gene set size of p = 200. The background colors refer to the time windows of phenotype response: immediate stress response (0–6 h), reprogramming decision (12–24 h), cell wall rebuilding and cell division (36–96 h). **B**, Whole transcriptome trajectory of the mutual information *I* versus Pearson correlation *r_V_*: time increases along the trajectory. The node annotation refers to the respective time in hours. Colors correspond to the transdifferentiation time phases as defined in **A. C**, Euclidean distance between correlation trajectories of ranked gene subsets and whole transcriptome. Dotted, colored lines depict the Euclidian distances from comparing whole transcriptome and ranked gene subsets for different gene subset sizes p =  [100,150,200,300,400,500,1000]. The red line depicts a least squares fit of a 7^th^ order polynomial to all data points. Dark and light grey areas mark the significance cutoffs of the MDS analysis (q-value <0.05) and transcriptome trajectory approach (rank 1,500). Black arrows indicate the five predicted TFs with putative involvement in transdifferentiation and the non-involved PpRSL2 paralog (Phypa_165193). The five small plots depict sample gene subset trajectories of the mutual information *I* versus the Pearson correlation *r_V_* (green lines). The black lines denote the whole transcriptome trajectory from **b**. The arrows mark the respective data points for the Euclidean distance (Eucl. Dist.) in the large plot.

Therefore, we defined a temporal state space trajectory of the transcriptome from its Pearson correlation and mutual information with respect to the 0 h time points [25] (Fig. 3A and B) and compared this trajectory with those of gene subsets: genes were ranked according to the associated p-values from the MDS analysis and then split into subsets of equal size (cf. Methods). For each gene subset, a correlation trajectory was calculated and compared to the global trajectory using the Euclidean distance in correlation space. We assume that subset trajectories following the whole transcriptome trajectory (small Euclidean distance and/or large correlation) contain genes contributing to the overall phenotypic response caused by detachment. Quantifying the contribution of each gene subset to the global gene response we found that moderately strong responding genes up to rank position 1,500 lie significantly closer to the global gene trajectory (Fig. S9), while the most differentially regulated genes do not follow the global change of the transcriptome (Fig. 3C). The calculation of the individual data points in Fig. 3C is depicted in the subpanels surrounding the large plot. Therein, the black lines show the global trajectory from Fig. 3B, which is the same for all plots. The green lines indicate the correlation trajectories for the specific gene subsets. Clearly, the correlation trajectories for the different gene subsets change. For genes with low rank numbers, gene subset and whole transcriptome trajectories are similar in shape and lie close to each other. However, trajectories differ from each other with increasing gene rank (top row of subplots) and consequently, the Euclidean distance between the green and the black trajectories increases. This way we define a set of 1,500 genes putatively controlling transdifferentiation, which includes phenotype related genes as well as 28 TFs (Fig. S5).

### TFs with unique kinetics are predicted to act as key players

**Figure 4:**
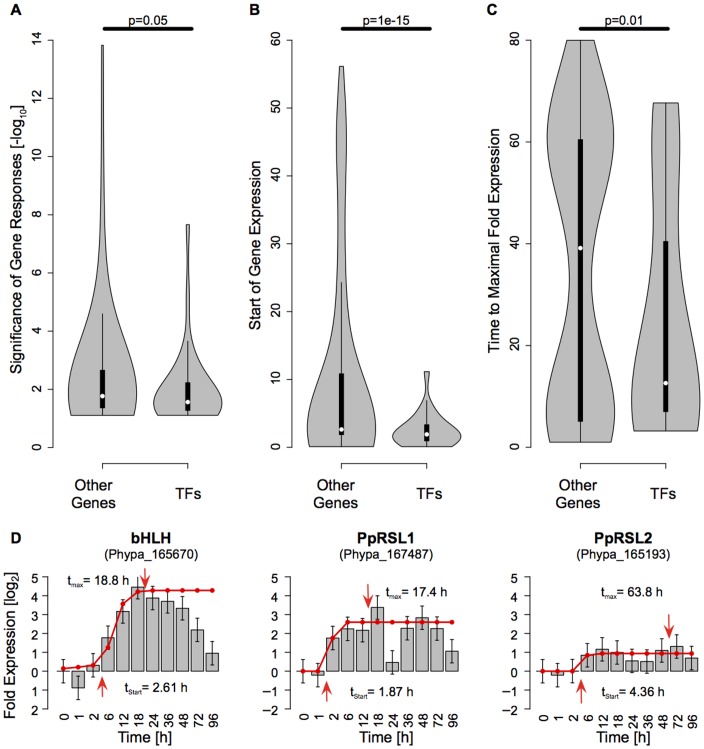
TF analysis and microarray-based fold expression profiles. Violin plots of differential gene response and gene kinetics for 1,500/28 genes/TFs (“Other Genes”/TFs) with predicted impact on transdifferentiation. **A,** p-value distribution of differential gene response kinetics. **B,** Start distribution of gene expression. **C,** Distribution of time from start to maximal fold change of gene expression. Vertical black bars, white dots and shaded areas denote the interquartile range, median values and kernel density, respectively. The p-values of a one-sided t-test denoting the significance of distribution differences are shown above the horizontal bars. **D,** Response kinetics of the bHLH TFs. Red lines denote a logistic fit to the expression kinetics. Arrows mark the onset and time of maximal fold expression. Error bars are estimated from the whole transcriptome standard deviation.

Characterizing the kinetics of these TF genes we find that both their start and their time to peak expression precedes that of other genes (Fig. 4B, C), although they are less strongly responding (Fig. 4A). The majority of TFs become maximally expressed within the first 24 h (Fig. 4C). Focusing on the hypothesized time window of the reprogramming decision 12–24 h a.d. (Fig. 1), only five genes show a specific up-regulation during this phase and closely follow the global transcriptome trajectory: two basic helix-loop-helix (bHLH; Fig. 4D), one APETALA2 (AP2), one mTERF and one MADS-box TF (Fig. S5).

One of the predicted bHLH TFs (PpRSL1, ROOT HAIR DEFECTIVE 6-like 1, Phypa_167487) has previously been described [26] as a positive regulator of caulonema and rhizoid development together with its functionally partially redundant paralog PpRSL2 (Phypa_165193; Table 1). The paralog pair has been argued to be functionally orthologous to the *Arabidopsis thaliana* ROOT HAIR DEFECTIVE 6 (RHD6) gene, a positive regulator of root hair development [26], [27]. In the phylogenetic tree (Fig. S6) the ortholog relationship between *A. thaliana* RHD6/RSL1 and *P. patens* RSL1/2 is clearly evident. However, in our analysis PpRSL2 is not predicted to be involved in the cellular decision due to the fact that it does not show predominant activation in the early phase alone (Fig. 4D) and is ranked beyond the first 1,500 genes (Fig. 3C). Estimation of up-regulation time of the two predicted bHLH TFs is 1.87 h and 2.61 h (Fig. 4D), maximum activation 17.4 and 18.8 h a.d., i.e. during the early phase (12–24 h a.d.; Fig. S7). The second bHLH factor (Phypa_165670) belongs to the same bHLH subfamily as RSL1 and 2, namely the RHD/RSL clade (Fig. S6, Table 1). PpRSL1 has previously been shown to be repressed by abscisic acid [28], suggesting a role in ABA/drought signaling. Given our transcriptome trajectory reconstruction (Fig. 3C, 4D), we hypothesized (i) that PpRSL1 would be involved in the establishment of apical stem cells and (ii) that PpRSL2 would not exert a critical influence on cell transdifferentiation after leaflet detachment. Therefore we analysed the *P. patens* RSL1/2 loss-of-function single (Δ*rsl1*, Δ*rsl2*) mutants [26] as well as the double mutant (Δ*rsl1/2*) with regard to leaflet regeneration.

**Table 1:** Main transcription factors discussed in the manuscript.

*P. patens* v1.2 protein id	Name	TF family	Predicted to be involved?	Loss-of-function mutant studied?	Comment
Phypa_167487	RSL1	bHLH	yes	yes	
Phypa_165193	RSL2	bHLH	no	yes	Close paralog of RSL1
Phypa_165670	n.a.	bHLH	yes	no	Same clade as RSL1 and 2

### Phenoytypic analyses of loss-of-function mutants confirm key player predictions

**Figure 5:**
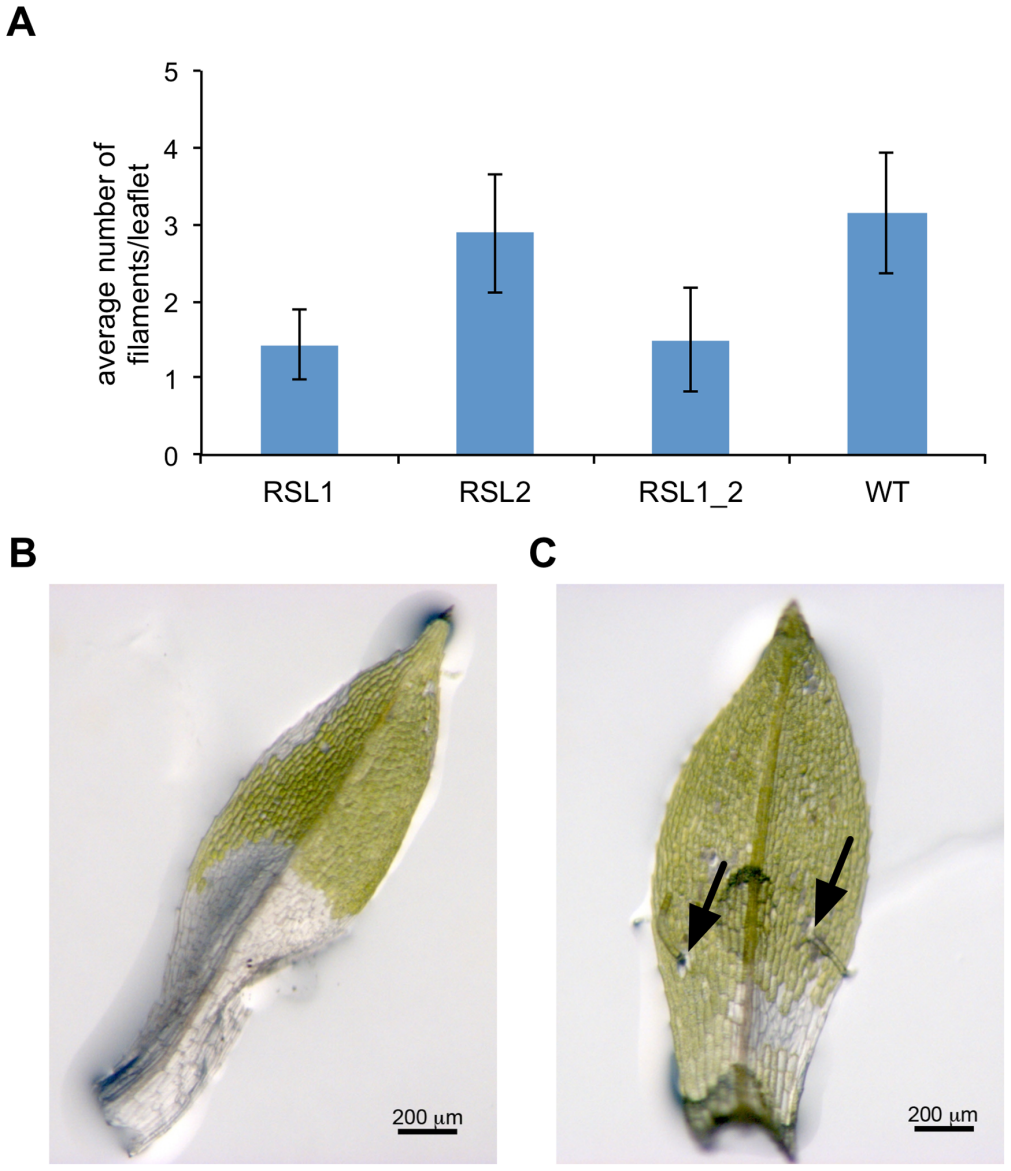
Phenotypic analysis. **A,** Average number of filaments per leaflet 72 h a.d. (error bars: standard error; RSL1&1_2 vs. RSL2&WT: p<0.05, one-sided t-test, n = 4–6). Detached leaflet of Δ*rsl1* (**B,** without filament) and wildtype (**C,** with two filaments protruding close to the wounding site; arrows) at 72 h a.d.

As a measure for transdifferentiation into apical stem cells we took the average number of filaments formed per leaflet 72 h a.d. That number was found to be significantly (p<0.05, one-sided t-test, n = 40–60) lower in Δ*rsl1* and Δ*rsl1/2* mutants as compared to the wildtype and Δ*rsl2* (Fig. 5A). No significant difference was detected between Δ*rsl2* and wildtype or between Δ*rsl1* and the double mutant. While the double mutant and Δ*rsl1* form 1.50 and 1.43 filaments per leaflet, respectively, Δ*rsl2* forms 2.88 and the wildtype 3.15 (Fig. 5). Since not all leaflets form filaments, we also used the number of leaflets that have formed at least one filament 72 h a.d. as an indicator. Here, we found that 37.5% of the double mutant and 43.3% of Δ*rsl1* leaflets have formed filaments, while this number was 61.7% for both Δ*rsl2* and the wildtype. We conclude that *RSL1*, with its distinct early activity peak around 18 h a.d. (Fig. 4D), is clearly positively involved in the transdifferentiation of leaflet cells into apical stem cells, while its close paralog *RSL2* is not. Thus, Δ*rsl1* as well as the double mutant exhibit a significantly reduced capacity to form filaments (Fig. 5A), which demonstrates a broader role than previously anticipated of the bHLH ortholog PpRSL1.

### The role of bHLH TFs in cellular decisions: influence of redundancy and dimerization?

Our approach singled out TFs putatively involved in the transdifferentiation process, which was confirmed in the case of PpRSL1. Namely, we find *PpRSL1* expressed early during the reprogramming, before the cells are transformed into apical cells and start their tip growth. Similar to this, the *AtRHD6* gene is expressed in hair cells of the root before they grow the protuberances that are known as root hairs [26]. It should be noted that the only other tip-growing cells in *A. thaliana*, pollen tubes, are not affected by down-regulation of AtRHD6 [26]. Therefore, the function of the *A. thaliana* protein has apparently been specialized during evolution, while the *P. patens* protein has kept a more general functionality, although it is still able to complement the Atrhd6 phenotype [26].

Constitutive expression of *PpRSL1* and *2* in *P. patens* leads to massive generation of rhizoids from gametophore tissue [29]. In both, *A. thaliana* and *P. patens*, only the double knockouts of the respective close paralog pairs severely affect root hair, respectively caulonema and rhizoid development, demonstrating that both paralogs are involved in positively regulating these cell types to start tip growing. It may well be that this is achieved by the two proteins acting as a heterodimer, as bHLH TFs are required to act as dimers.

With regard to the reprogramming of differentiated leaflet cells into tip growing chloronemal cells in *P. patens*, however, it is tempting to speculate that instead of PpRSL2, which we have shown not to contribute to this process, the other bHLH subfamily member identified by us (Phypa_165670) will form a heterodimer with PpRSL1 in order to achieve this particular regulation (Fig. S7). In this context, comparable signaling by bHLH heterodimers has been described in other systems, e.g. the human HES/HERP proteins, which are Notch effectors and affect cell fate decisions via repression of downstream target genes [30]. This suggests that some differentiation processes in complex animals and plants might follow the same underlying signaling principles.

Since redundancy of the PpRSL1/2 paralogs in formation of rhizoids from gametophore stems *vs*. chloronemal filaments from leaflets is different, we looked into another transdifferentation process, namely the formation of vegetative diaspores, brachycytes, from protonemal cells. Here, all three mutants are severely impaired with regard to number of brachycytes formed (Table S4). Also, the double mutant formed only 8% of subapical brachycytes, while it generated 78% of apical brachycytes. Hence, the formation of brachycytes, with regard to functional redundancy of both paralogs, is more akin to rhizoid formation than to apical stem cell formation from detached leaflets, where only PpRSL1 is involved.

Interestingly, coupling of environmental and developmental cues via cascades of bHLH proteins occurs in many systems from human neurons to seed plant root hairs [31]. Here, we find similar evidence for bHLH cascading, as bHLH TFs appear highly ranked in all phases of the reprogramming (Fig. S7). Most probably, bHLH TFs act together with further players as described in e.g. neuron development, where an interplay of bHLH and homeodomain TFs is required for cell fate specification [32].

### Summary and outlook

Time-resolved gene expression kinetics are able to reveal details about the molecular and developmental time-sequential pattern of developmental progression in complex systems; here: *in vivo* apical stem cell formation in *P. patens* upon detachment of leaflets over a time course of several days. We introduce a novel methodology to predict key players from time-resolved transcriptome data by (i) defining genes that follow the global transcriptome response and are moderately regulated and (ii) isolating TFs among this population that display peak expression in the critical phase (here: reprogramming of a leaflet cell into an apical stem cell). The present analysis of transcriptome trajectories singled out several TFs, predominantly from the bHLH family, putatively involved in transdifferentiation (Fig. S5). Phenotypic analysis of loss-of-function mutants demonstrates that the prediction is valid and that the closely related paralogs PpRSL1 and 2 do not act redundantly with regard to establishment of apical stem cells. We expect the methodology introduced here to aid in the detection of synergistic action of TFs involved in developmental processes of complex systems. In particular, it might be employed to predict key regulators during aberrant gain of pluripotency by cancer cells. Future analyses of this kind in different systems might reveal general principles of networks controlling developmental progression in plants and animals.

## Materials and Methods

### Tissue culture and microscopy

*Physcomitrella patens* Gransden 2004 [33] was grown as previously described [13]. Preculture of gametophores was carried out by placing single gametophores with their stem into agarized (solid) medium and subsequent growth for four weeks. Leaflets were detached using forceps and placed into liquid medium or onto solid medium covered by a sheet of cellophane. Detachment of leaflets was always carried out in the middle of the 16 h light period and detached leaflets were subsequently placed under the same 16 h light/8 h dark regime under which the preculture had occurred. Therefore, the 12 and 36 h a.d. harvest time points represent the dark period and all other time points the light period. Harvesting of leaflets was carried out by filtering through nylon mesh followed by immediate freezing in liquid nitrogen.

### Phenotypic analyses

Detached leaflets were observed under a stereo microscope 72 h after detachment (a.d.) in order to determine the number of filaments per leaflet. Four to six repetitions with 10 leaflets each were carried out. For setup and long term observation refer to Fig. S8. To determine the influence of osmotic conditions, leaflets were incubated in liquid mineral medium vs. iso-osmotic protoplast regeneration medium supplemented with glucose and mannitol to 540 mOs. Three repetitions with 10 leaflets each were carried out and analyzed 120 h a.d. Brachycytes (vegetative diaspores) were induced by adding (+)-*cis*, *trans*-abscisic acid (ABA; Duchefa; 25 µmol final concentration) to freshly homogenized protonemal liquid culture set to 100 mg*L^−1^ dry weight. Observation was carried out after two weeks and formed brachycytes scored into apical, subapical or side-branching, respectively. Six repetitions were carried out and total number of scored brachycytes was: WT 315, *rsl1* 222, *rsl2* 280, *rsl1/2* 161. For number of brachycytes formed, 100 µL culture per line was scored in three independent replicates.

### RNA Isolation

Isolation of total RNA was performed using the RNeasy Plant Micro Kit (Qiagen, Hilden, Germany) with an on-column DNase treatment following the manufacturers’ protocol. Using the MessageAmp II aRNA Kit (Ambion, Texas, USA), 100 ng total RNA were amplified to yield sense-strand amplified RNA (aRNA), which was reverse transcribed into first strand cDNA using SuperScript III (Invitrogen, Karlsruhe, Germany) and random hexamer oligonucleotides (Fermentas, St. Leon-Rot, Germany).

### Microarray experiments

Microarray experiments (including RNA isolation, amplification and labeling) were carried out as previously described [13]. Due to the low amount of available material, two subsequent rounds of amplification were carried out, according to the manufacturers' protocol (MessageAmp II aRNA Kit; Ambion, Texas, USA). All RNA samples were quality checked using a Bioanalyzer 2100 (Agilent, Santa Clara, USA). Raw data processing was carried out using Analyst 2.0 (Genedata, Basel, Switzerland) as previously described [13]. Probe sets were normalized (median scaling to a target value of 1) using a rank invariant set (RIS), which consisted of 48 non-differentially expressed genes (fold change <1.25) common to all pairwise array-to-array comparisons. The resulting 27,715 probes were further filtered prior to analysis. Lowly expressed genes (abundance values <1 for more than 75% of all time points per gene) and genes with an inter quartile range <0.25 were discarded, reducing the filtered set to 17,158 probes. Fold expression values were calculated with respect to the mean of the 0 h time point, taken in duplicate and log2 transformed.

### Quantitative realtime PCR

The EMBOSS implementation eprimer3 (http://emboss.sourceforge.net/) was used for design of gene-specific oligonucleotides (Table S2). Quantitative realtime PCR was performed using SensiMix SYBR Kit (Quantace, Berlin, Germany) on a LightCycler 480 (Roche, Mannheim, Germany) according to the manufacturers’ instructions. For each 25 µL reaction 50 ng of aRNA-equivalent were used. Triplicate measurements on each sample and melting curve analysis were performed for all samples to ensure product specificity. Results from reactions with multiple products were removed prior to further analysis. Resulting Cp-values (crossing point, threshold cycle) were normalized to 60S ribosomal protein L19 (Phypa_222528) and ribosomal protein S21 (Phypa_61453), employing the comparative Ct method (ΔΔCt), as their measured expression levels showed the lowest variation between the time points used. qPCR validation at 24 h a.d. was carried out on an independent biological replicate to confirm the up-regulation of the three TFs predicted to be involved and FIE (Phypa_61985). All four genes showed high log2 fold changes at 24 h a.d. relative to time point zero (Table S3).

### Periodicity analysis

Periodic patterns in time series resulting from biological experiments are of great interest. Direct Fourier analysis is only applicable when data points are evenly spaced, which is not the case in our experimental setup. The Lomb – Scargle periodogram approach can quantify the periodic behavior of the gene expression time series for every gene. This analysis provides a direct method to treat unevenly spaced time points. Here, the method proposed by [18] is used, which combined a Lomb – Scargle test statistic for periodicity with a multiple hypothesis testing procedure to detect significant periodic gene expression patterns. We tested 1,500 regulated genes that were predicted to be important in transdifferentiation for dominant oscillation periods *T_Gene_* around 24 h (Fig. 2). We used the time points from 0–72 h for periodogram analysis, such that the critical period of 24 h lies well between the lower and the upper frequency bounds of the spectral analysis. Based on the mean time interval of the 10 measured time points, the periodogram was evaluated at 40 evenly spaced test frequencies between 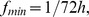 and 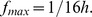 88 genes show a dominant oscillation period 23 h<P<25 h, with the lowest p-value (FDR-corrected q-value) of 0.07 (0.605). Therefore circadian rhythmicity does not seem to have a dominant role in this data set.

### Significance analysis of gene subset and whole transcriptome distance

To assess the significance of the local minimum of the Euclidean distance between moderately regulated genes and the global transcriptome trajectory, we compared the goodness of fit of polynomial regression models to the set of Euclidean distances from rank ordered or randomly ordered gene subsets. For this, we fitted the data points from the ordered and the randomized subsets to both a full and a reduced model and calculated the deviation from the ratio of the likelihood ratio statistic and the likelihood under the full model [34]. In detail, we fitted a 7th order polynomial jointly to the data points from ordered and randomized gene subsets
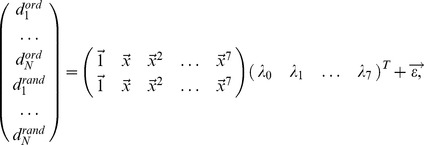
where 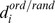 denote the Euclidean distance from the ordered/random gene subsets to the whole transcriptome trajectory. 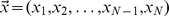 and 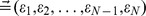 denote the mean rank of the residual error term, respectively. N = 520 is the total number of gene sets with different size p =  [100, 150, 200, 300, 400, 500, 1,000] considered. The parameters 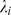 denote the model coefficients. We compared the goodness of fit of the reduced model above to a full model that allows separate coefficients for the randomized and ordered gene sets.



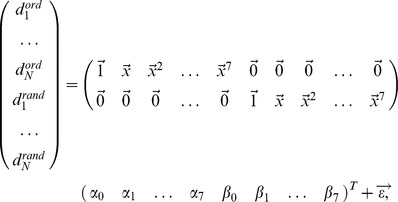
where the meaning of the variables and coefficients 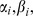 remains the same. If the regression model using the ordered gene subsets is significantly different from the one obtained using a randomized gene list, the residual sum of squares should become substantially smaller when fitting the full model with more parameters as compared to a fit with the reduced model. Hence, we propose the null hypothesis that the quality of fit does not change when fitting the ordered and the randomized data either to the full or the reduced model. If that is the case, the minimum of the Euclidean distance between moderately regulated genes and the global transcriptome trajectory cannot be explained from the specific gene response strength. The difference between the deviances for the two models follows approximately a chi-square distribution with k degrees of freedom, where k is the number of additional parameters available to the full model, from which we calculated the p-value using the ANOVA function in R. Testing different subset sizes and all data points from all subsets together, we always found p-values <1e–5 (Fig. S9), indicating that the shape of the fitted polynomial is indeed a result of using an ordered list of genes, confirming the local extrema for minimal distance and maximal correlation of moderately strong responding genes.

### Ranking and multi-dimensional scaling

Dynamic gene response was estimated from a multi-dimensional scaling analysis (MDS), which projects the distance matrix containing the Euclidian distances between all gene kinetics onto a two dimensional space. To calculate the projection, retaining the original distances as closely as possible, we applied the HiT-MDS algorithm [35], which maximizes the Pearson correlation of the original and the reconstructed distances using a gradient descent approach. The resulting point distribution was then fitted to a skewed multivariate normal distribution using the R-package ‘sn’ [36] (Version 2.14, http://www.R-project.org).

P-values 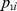 for each gene, denoting the gene response, are calculated from the integral over the normed probability density of the skewed multivariate normal distribution
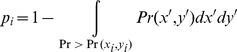
where 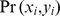 denotes the probability density of gene *i* located via the MDS projection at 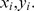 Genes are subsequently ranked according to their false-discovery rate adjusted q-values applying the Benjamini-Hochberg procedure [37].

### Gene Ontology analysis

Gene Ontology (GO) analysis was performed using the GOstats library from R (Version 2.14, http://www.R-project.org). The *P. patens* GO annotation (11,328 annotated genes) was used [33]. Bias analysis of GO terms related to biological process (BP) was done via a conditional hypergeometric test using a cutoff of p<0.05.

To robustly estimate differentially regulated genes per time point in the commonly encountered presence of outliers and skewness [38], we fit a skew t-distribution distribution [36] to the log_2_ fold change between the expression values at 0 h and the respective time points (Fig. S10). In addition to the regular t-distribution 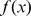 with 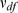 degrees of freedom, a skew-t distribution, 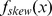 has an additional skewing parameter 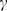 such that
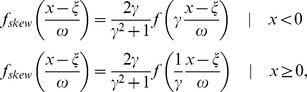
where 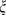 and 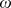 denote the location and scale of the distribution.

The gene fold expression histogram was fitted using maximum likelihood estimation for the univariate skew-t distribution, implemented in the R package ‘sn’. Maximum likelihood estimation of the distributions was in good agreement with the experimental data outperforming other approaches such as robust estimation (Fig. S3). Regulated genes having a p-value <0.05, one-sided t-test, were considered significantly up- or down-regulated (Fig. S3, S10).

### Whole transcriptome and gene subset correlation trajectories

Transcriptome trajectories are visualized in a two-dimensional correlation space defined by the Pearson correlation and mutual information of the temporal gene deviations [25]. As the linear correlation measure we use a modified Pearson correlation.
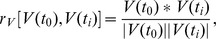
where the vectors 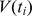 and 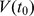 contain the deviations of each gene from their temporal mean at the time points *t_i_* and *t_0_*. *r_v_* accounts for differences in expression variability, yet similar temporal profiles. The mutual information



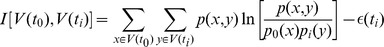
accounts for non-linear relationships between two gene deviation vectors 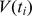 and 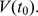 The joint and marginal distributions *p(x,y)* and *p_0_(x)*, *p_i_(y)* are estimated by discretizing the gene expression deviation. The constant 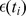 corrects for the systematic error from discretization of the continuous gene expression values and is calculated from the minimal *I* using 100 random permutations of 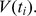


To calculate the whole transcriptome Pearson correlation *r_V_* and mutual information *I* between time points t_0_ and t_i_, we generated the vectors *V_0_* and *V_i_* from sampling gene sets of size *p^transcriptome^* from the whole transcriptome (N = 17,158) and taking the average over 1,000 repeats. Plotting 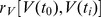 against 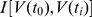 for all time points defines a trajectory in correlation space for the whole transcriptome. To assess the contribution of individual gene sets to the dynamic behavior of the whole transcriptome, we first ranked all genes according to the MDS p-values. The ranked list of N = 17,158 genes was then divided into non-overlapping gene sets of equal size *p^subset^*. For each of these gene groups we calculated a subset trajectory and compared each trajectory to the whole transcriptome trajectory of the same sampling size (*p^subset^* =  *p^transcriptome^*) using the Euclidean distance as well as the Pearson correlation (Fig. S11). We assume that subset trajectories having a small Euclidean distance and a large correlation with the whole transcriptome trajectory contain genes that are important for the overall phenotypic development. The analysis was repeated for different set sizes, which we chose as *p* =  [*100,150,200,300,400,500,1000*], being limited by sampling noise and by resolution for small and large sampling set sizes, respectively.

### Estimation of gene induction times

Gene induction times were estimated by approximating the mRNA fold expression values to a logistic function [39] 
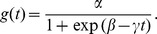


Parameter fitting for 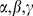 was done using a Levenberg-Marquardt non-linear least squares algorithm as implemented in the R package minpack.lm. The up-regulation time for each gene was defined as the time of maximal change in the acceleration of the logistic function. This point can be calculated by finding the maximum of the third derivative (i.e., the first time at which the fourth derivative of the logistic function equals zero).

### Data access

The microarray data can be accessed from ArrayExpress (http://www.ebi.ac.uk/arrayexpress/) under the accession number E-MTAB-915.

## Supporting Information

Figure S1 **Mean and variance of transcriptome response** ***in P. patens*** **after leaflet detachment. A**, Mean and **B** standard deviation of leaflet transcriptome fold change a.d. Two independent experiments were conducted, labeled S and T; the 0 h time point was sampled in both. The fold change has been normalized with respect to the two experiments performed individually at 0 h (shown in blue and black, respectively), as well as to their mean (shown in red).
(PDF)

Figure S2 **p-value histogram of transcriptome response and multi-dimensional scaling of the gene expression profiles up to 96 h a.d. A**, p-value histogram for dynamic transcriptome response scores evaluated from the MDS analysis and fitting with a bivariate skew normal distribution, resulting in a long-tailed distribution. The barplot insert shows the log2 fold change dynamics of two previously known markers for apical stem cell differentiation, the transcriptional regulator PpFIE (Phypa_61985) as well as Cyclin D;1 (Phypa_226408). **B**, Multi-dimensional scaling (MDS) analysis of transcriptome response to leaflet detachment using 17,128 genes [35]. Symbol colors indicate the p-value of differential regulation for the whole time course. Significantly regulated genes with a FDR-corrected q-value <0.05 are marked by black dots and are additionally labeled with the *P. patens* gene IDs. The positions of the five predicted TFs as well as the weakly regulated PpRSL2 (165193) are additionally indicated by red squares. The red dashed lines mark curves of equal probability density by fitting a bivariate skew normal distribution to the point distribution.
(PDF)

Figure S3 **Gene Ontology analysis of transcriptome response after leaflet detachment.** Biased biological process GO categories for significantly regulated genes at 1–96 h a.d. Gene fold expression profiles were fit to a skew-t distribution and considered significantly up- or down-regulated for a p-value <0.05. GO analysis was done using a conditional hypergeometric test from the R Bioconductor package GOstats, using as background 11,283 genes having a GO annotation. GO categories were considered significant with a cutoff p-value <0.05. The p-values in the plot are log-transformed and color-coded in a range from 0 to 5. Highlighted categories are mentioned in the text.
(PDF)

Figure S4 **Protonemal filaments occasionally emerge from leaflets of slowly dehydrated gametophores.** Gametophores were slowly dehydrated over several weeks in petri dishes devoid of covering laboratory film. Under these conditions, occasional emergence of protonemal filaments from leaflets of dehydrated gametophores can be seen (arrows).
(PDF)

Figure S5 **Transcription factors among the top ranked genes.** TFs putatively involved in the development of apical stem cells in *P. patens*. All 28 annotated TFs [40] up to gene rank 1,500 are shown. Colored backgrounds indicate a potential role in differentiation due to their dominant peak in fold expression within the early phase (12–24 h a.d.) and ranking among the first 1,500 genes. The green highlighted TF was verified as involved in this study, the TFs in yellow were also detected based on their significant up-regulation in the early phase (cf. Figs. 3, S11). Error bars have been estimated from the variance of expression for the 0 h time point that was taken in duplicate.
(PDF)

Figure S6 **Phylogenetic tree of bHLH transcription factors.** Gene family tree of part of the plant bHLH proteins, centered on the RSL subclade (members shown in red). The tree was calculated based on bHLH protein domains using Bayesian inference as previously described [28], posterior probabilities are shown at the nodes. Accession numbers, resp. gene names for previously annotated *P. patens* and *A. thaliana* bHLH proteins, are shown. Besides proteins from these two organisms, OsBP-5 (CAD32238) from *O. sativa* was included, as it belongs to this subfamily. The two *P. patens* bHLH TFs detected by their early peaking upon leaflet detachment are PpRSL1 and Phypa_165670 (marked in blue), while PpRSL2 was shown not to be involved in apical stem cell formation (see text).
(PDF)

Figure S7 **Timeline of transcriptional activation after detachment.** Mean TF activity per time point, the three response intervals are denoted as well as TFs (shown by family assignment) significantly differentially regulated in the respective interval. Mean fold change denotes the sum of log2 fold change values of all annotated TFs [40] within the *P. patens* genome. The error bars denote the standard deviation of the mean fold change.
(PDF)

Figure S8 **Long-term observation of detached leaflets.** Petri dish of a leaflet detachment/transdifferentiation experiment, 62 d a.d., demonstrating the lack of rhizoids in the double mutant (upper right) and that no severe differences in long term gametophore growth are visible (wt: upper left; Δ*rsl1* lower right; Δ*rsl2* lower left).
(PDF)

Figure S9 **Regression Analysis of Gene subset Euclidean Distance.** The Euclidean distances of ordered (dark red lines) and randomized (grey lines) gene subsets for different gene set sizes and all gene sets combined have been fitted with two different polynomial regression models. Plot titles denote the respective gene set size. A full regression model allows separate fitting of the randomized and ordered gene sets, depicted by the black and orange dashed lines, respectively. A reduced model (red dashed line) fits both types of gene subsets simultaneously. The p-values of the ANOVA comparison of the two models confirm the differences between the full and reduced model. Thus, the local minimum of the Euclidean distance between moderately regulated genes and the global transcriptome trajectory is indeed a result of using an ordered list of genes.
(PDF)

Figure S10 **Skew-t distribution fit to gene fold expression. A, B** Normal, robust and skew-t distribution fit to the log2 gene fold change at 1 h (**A**) and 96 h (**B**). **C, D** Comparison of goodness-of-fit of the fitting distributions: quantile-quantile plots of the sample distributions. The skew-t distribution shows the best fit to the distributions, in particular with respect to the outliers at low/high quantiles. Abbreviations: SD: standard deviation, IQR: inter quartile range.
(PDF)

Figure S11 **Euclidean Distance and Pearson Correlation of Gene subsets and whole transcriptome trajectory. A**, Euclidean distance and **C** Pearson correlation of ranked gene subsets with respect to the whole genome trajectory. Both measures have their absolute maximum around gene rank 1,500. **B**, Projection of the Euclidean distance and Pearson correlation showing the simultaneous extremum of both measures for genes ranked between 1,000–1,500. The dark and light shaded areas in **a** and **c** mark the cutoff for significantly regulated genes from the MDS and transcriptome trajectory at rank 299 and 1,500, respectively. The cross in **B** marks the rank 1,500. The arrows in **A** and **C** depict the location of the five predicted TFs and of the non-involved paralog, as shown in Fig. 3.
(PDF)

Table S1 **Gene Ontology categories of biological processes being under-represented among the significantly regulated genes.** Significantly regulated genes as detected from the MDS analysis (299 genes with a q-value <0.05). Altogether, 11,283 genes were annotated and used as background. GO categories were considered significant with a cutoff of p<0.05.
(PDF)

Table S2 **Oligonucleotides used for realtime PCR.**
(PDF)

Table S3 **qPCR validation.** Log2-fold expression values relative to time point zero are shown.
(PDF)

Table S4 **Brachycyte formation upon ABA treatment.**
(PDF)
