# Supplementary figures and images for: Network Theory Inspired Analysis of Time-Resolved Expression Data Reveals Key Players Guiding P. patens Stem Cell Development
Source: PLoS One. 2013 Apr 18;8(4):e60494. doi: 10.1371/journal.pone.0060494 (PMC3630159; doi:10.1371/journal.pone.0060494)

**Fig. S1.** Mean and variance of transcriptome response in *P. patens* after leaflet detachment.

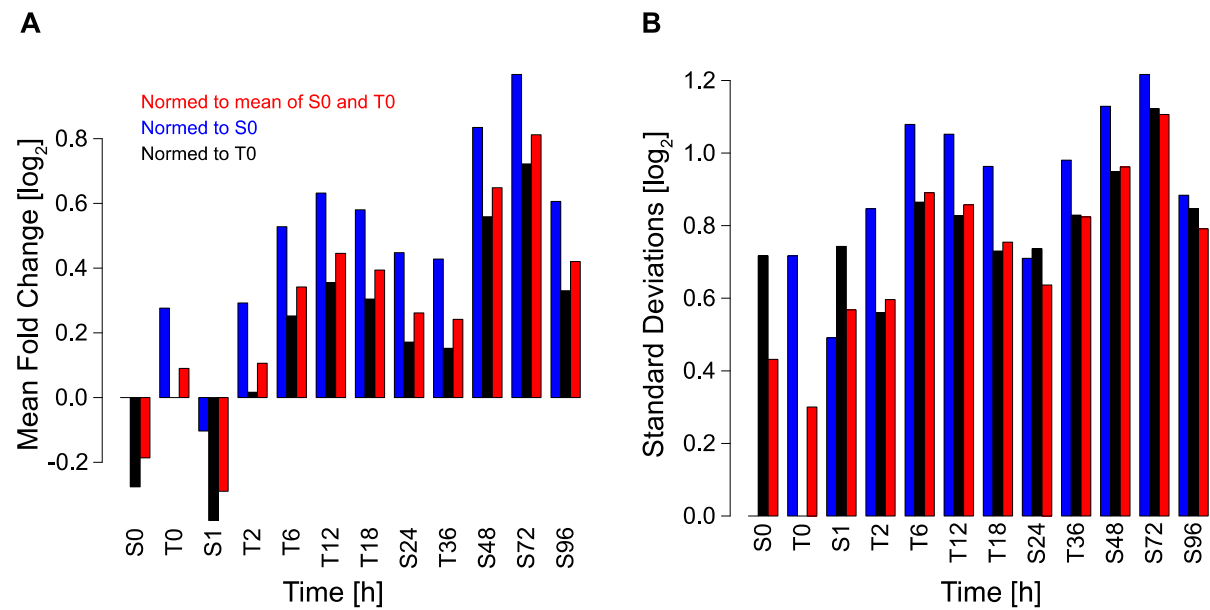

Supplement: Figure S1 — Mean and variance of transcriptome response in P. patens after leaflet detachment. A, Mean and B standard deviation of leaflet transcriptome fold change a.d. Two independent experiments were conducted, labeled S and T; the 0 h time point was sampled in both. The fold change has been normalized with respect to the two experiments performed individually at 0 h (shown in blue and black, respectively), as well as to their mean (shown in red). (PDF) [file pone.0060494.s001.pdf]

**Fig. S3. Gene Ontology analysis of transcriptome response after leaflet detachment.**

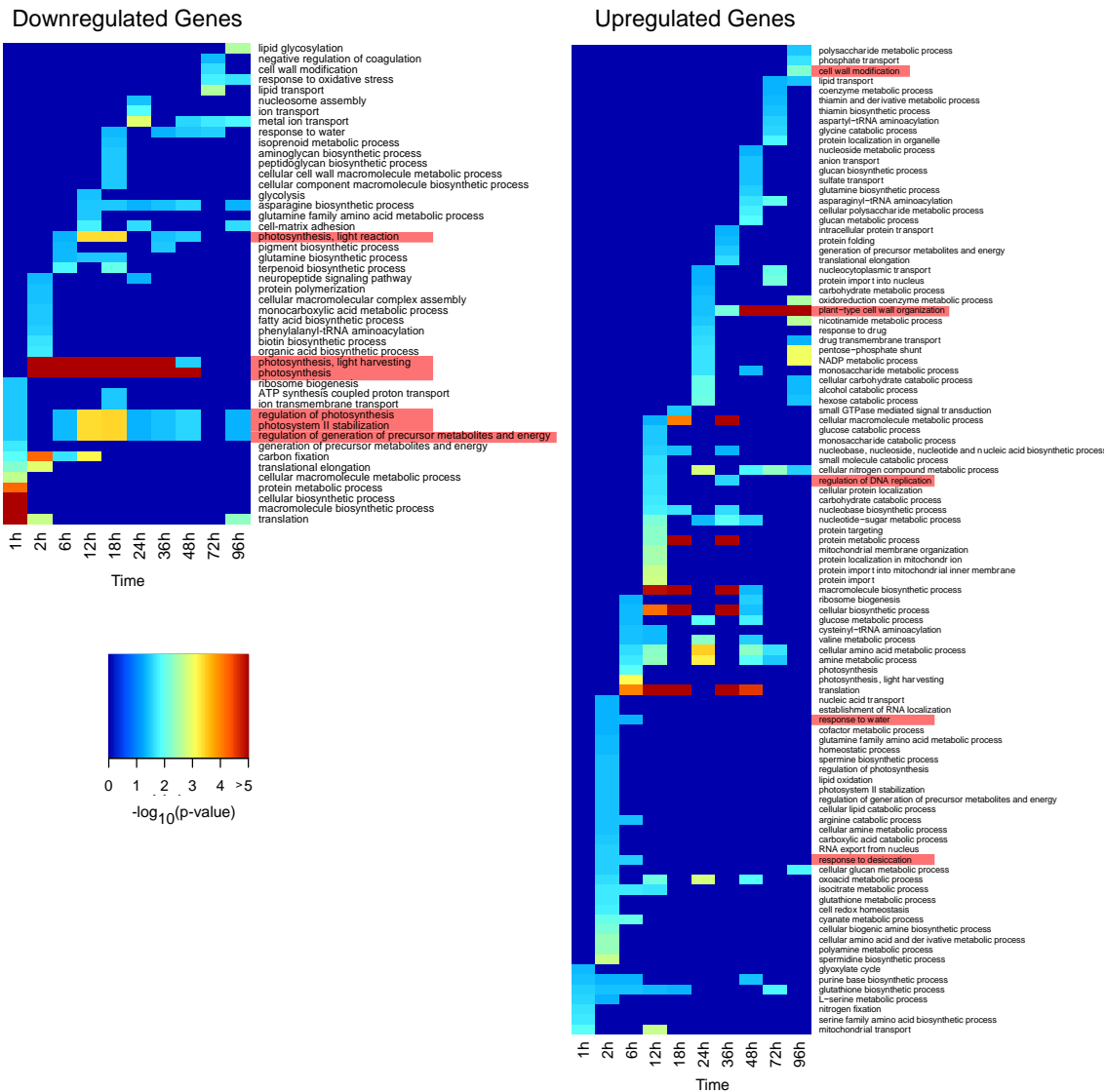

Supplement: Figure S3 — Gene Ontology analysis of transcriptome response after leaflet detachment. Biased biological process GO categories for significantly regulated genes at 1–96 h a.d. Gene fold expression profiles were fit to a skew-t distribution and considered significantly up- or down-regulated for a p-value <0.05. GO analysis was done using a conditional hypergeometric test from the R Bioconductor package GOstats, using as background 11,283 genes having a GO annotation. GO categories were considered significant with a cutoff p-value <0.05. The p-values in the plot are log-transformed and color-coded in a range from 0 to 5. Highlighted categories are mentioned in the text. (PDF) [file pone.0060494.s003.pdf]

**Fig. S5.** Transcription factors among the top ranked genes.

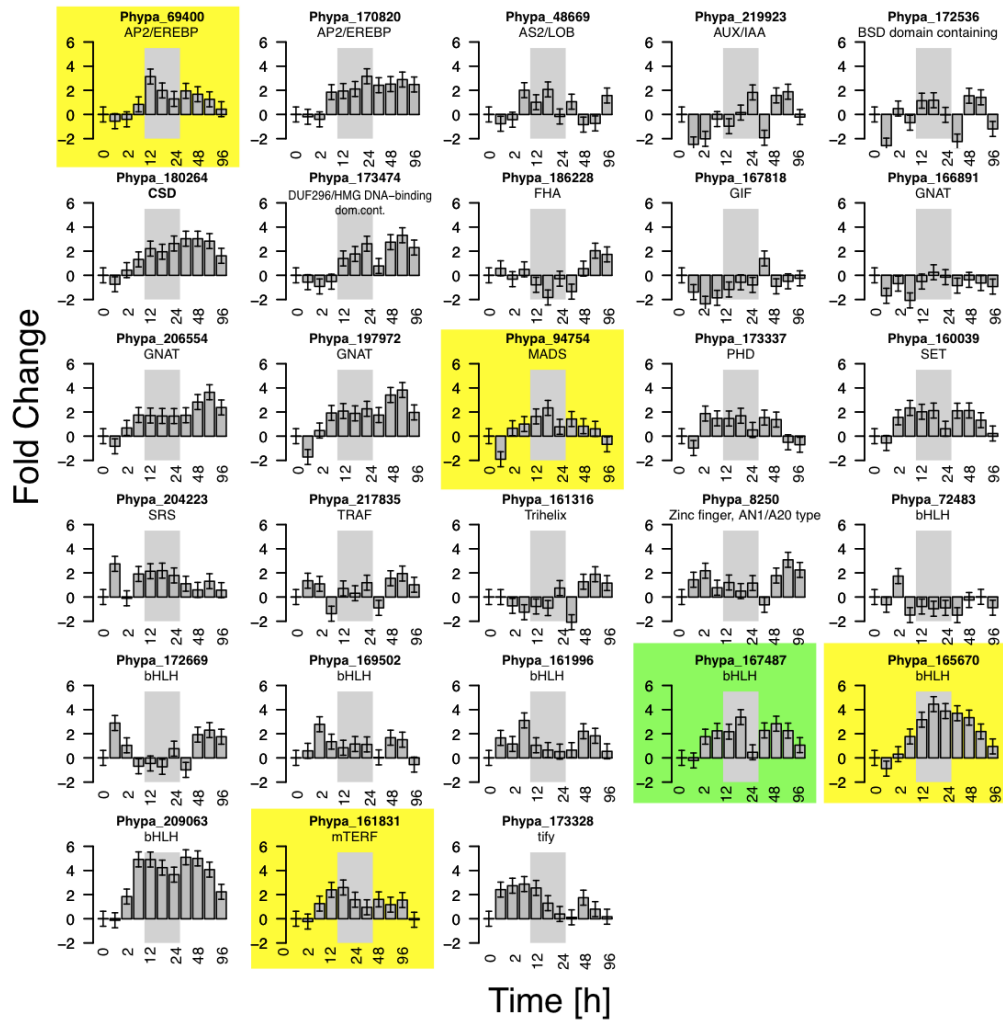

Supplement: Figure S5 — Transcription factors among the top ranked genes. TFs putatively involved in the development of apical stem cells in P. patens. All 28 annotated TFs [40] up to gene rank 1,500 are shown. Colored backgrounds indicate a potential role in differentiation due to their dominant peak in fold expression within the early phase (12–24 h a.d.) and ranking among the first 1,500 genes. The green highlighted TF was verified as involved in this study, the TFs in yellow were also detected based on their significant up-regulation in the early phase (cf. Figs. 3, S11). Error bars have been estimated from the variance of expression for the 0 h time point that was taken in duplicate. (PDF) [file pone.0060494.s005.pdf]

**Fig. S6.** Phylogenetic tree of bHLH transcription factors

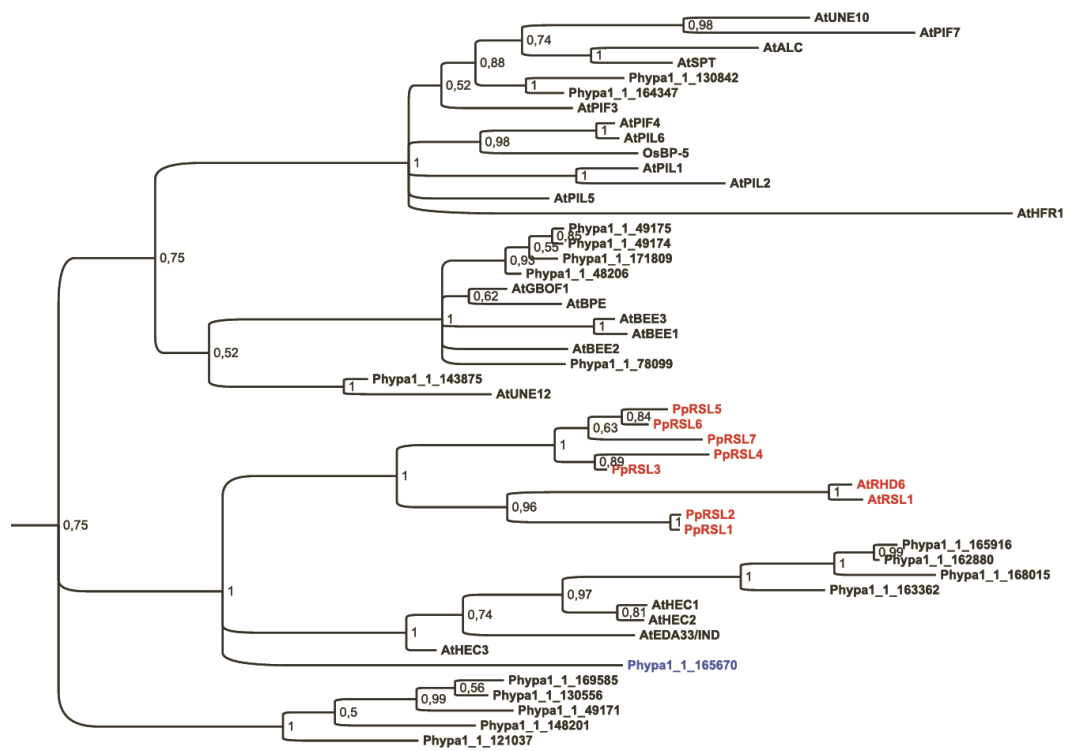

Supplement: Figure S6 — Phylogenetic tree of bHLH transcription factors. Gene family tree of part of the plant bHLH proteins, centered on the RSL subclade (members shown in red). The tree was calculated based on bHLH protein domains using Bayesian inference as previously described [28], posterior probabilities are shown at the nodes. Accession numbers, resp. gene names for previously annotated P. patens and A. thaliana bHLH proteins, are shown. Besides proteins from these two organisms, OsBP-5 (CAD32238) from O. sativa was included, as it belongs to this subfamily. The two P. patens bHLH TFs detected by their early peaking upon leaflet detachment are PpRSL1 and Phypa_165670 (marked in blue), while PpRSL2 was shown not to be involved in apical stem cell formation (see text). (PDF) [file pone.0060494.s006.pdf]

**Fig. S7.** Timeline of transcriptional activation after detachment.

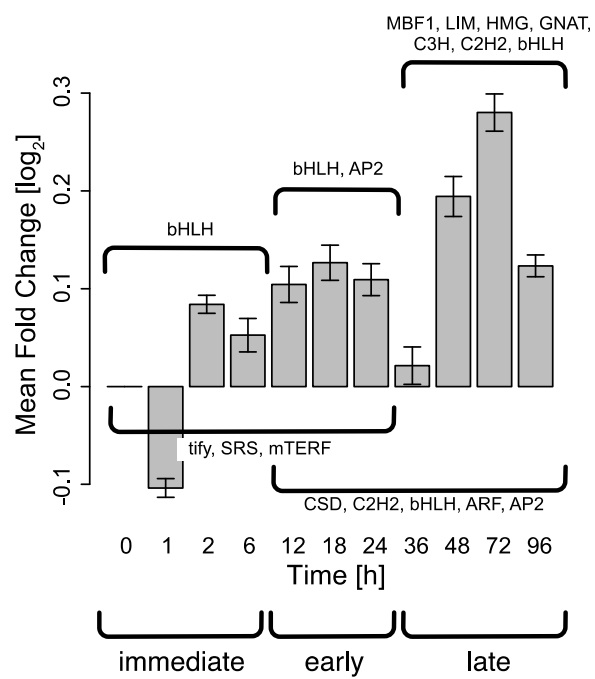

Supplement: Figure S7 — Timeline of transcriptional activation after detachment. Mean TF activity per time point, the three response intervals are denoted as well as TFs (shown by family assignment) significantly differentially regulated in the respective interval. Mean fold change denotes the sum of log2 fold change values of all annotated TFs [40] within the P. patens genome. The error bars denote the standard deviation of the mean fold change. (PDF) [file pone.0060494.s007.pdf]

**Fig. S8.** Long-term observation of detached leaflets.

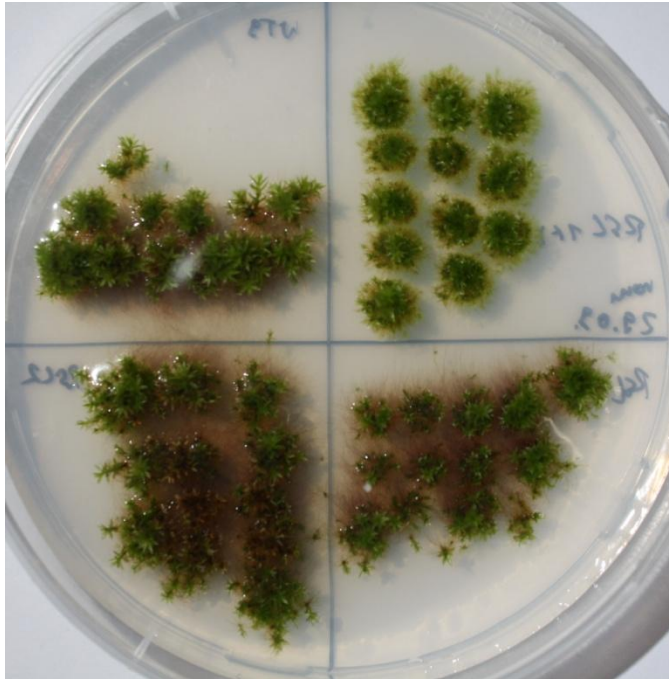

Supplement: Figure S8 — Long-term observation of detached leaflets. Petri dish of a leaflet detachment/transdifferentiation experiment, 62 d a.d., demonstrating the lack of rhizoids in the double mutant (upper right) and that no severe differences in long term gametophore growth are visible (wt: upper left; Δrsl1 lower right; Δrsl2 lower left). (PDF) [file pone.0060494.s008.pdf]

**Fig. S9.** Regression Analysis of Gene subset Euclidean Distance

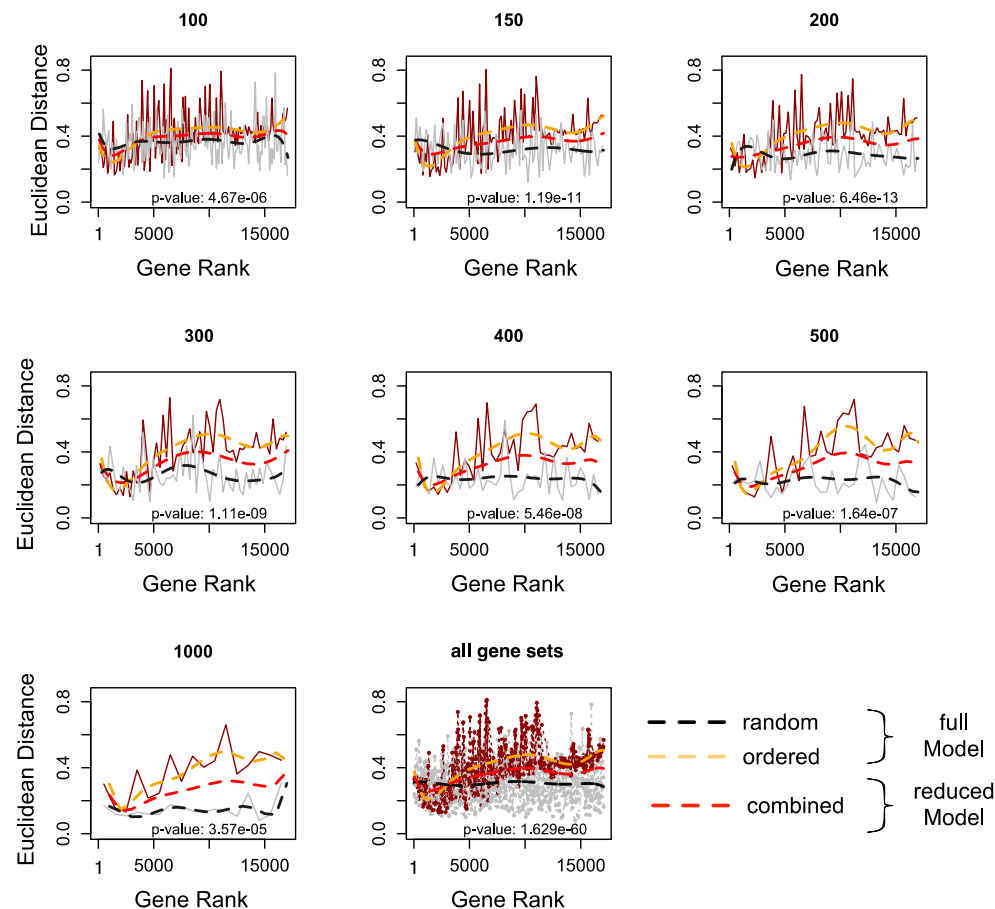

Supplement: Figure S9 — Regression Analysis of Gene subset Euclidean Distance. The Euclidean distances of ordered (dark red lines) and randomized (grey lines) gene subsets for different gene set sizes and all gene sets combined have been fitted with two different polynomial regression models. Plot titles denote the respective gene set size. A full regression model allows separate fitting of the randomized and ordered gene sets, depicted by the black and orange dashed lines, respectively. A reduced model (red dashed line) fits both types of gene subsets simultaneously. The p-values of the ANOVA comparison of the two models confirm the differences between the full and reduced model. Thus, the local minimum of the Euclidean distance between moderately regulated genes and the global transcriptome trajectory is indeed a result of using an ordered list of genes. (PDF) [file pone.0060494.s009.pdf]

**Fig. S10.** Skew-t distribution fit to gene fold expression.

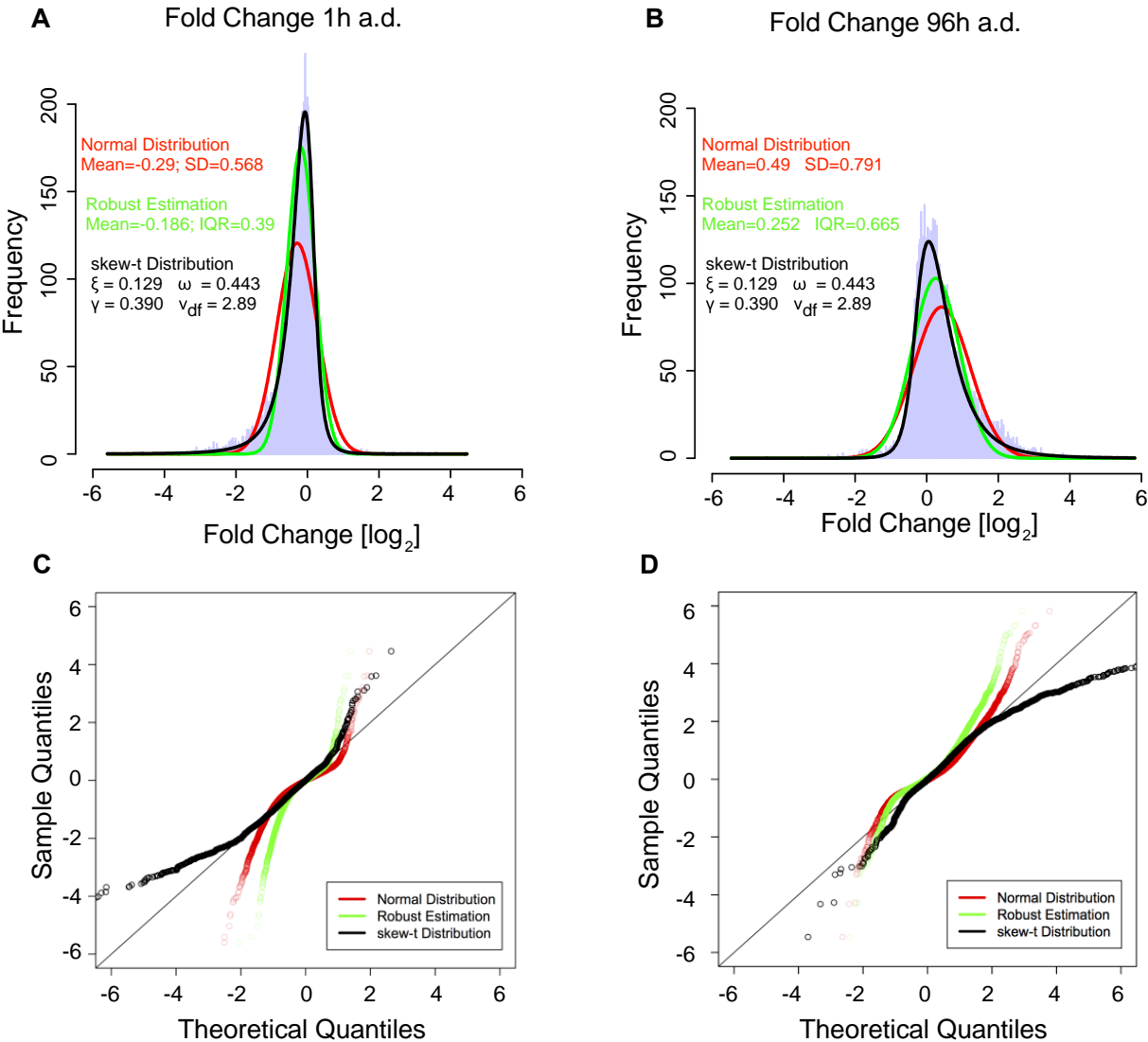

Supplement: Figure S10 — Skew-t distribution fit to gene fold expression. A, B Normal, robust and skew-t distribution fit to the log2 gene fold change at 1 h (A) and 96 h (B). C, D Comparison of goodness-of-fit of the fitting distributions: quantile-quantile plots of the sample distributions. The skew-t distribution shows the best fit to the distributions, in particular with respect to the outliers at low/high quantiles. Abbreviations: SD: standard deviation, IQR: inter quartile range. (PDF) [file pone.0060494.s010.pdf]
