# Supplementary material for: Network Theory Inspired Analysis of Time-Resolved Expression Data Reveals Key Players Guiding P. patens Stem Cell Development
Source: PLoS One. 2013 Apr 18;8(4):e60494. doi: 10.1371/journal.pone.0060494 (PMC3630159; doi:10.1371/journal.pone.0060494)

Fig. S2 p-value histogram of transcriptome response and multi-dimensional scaling of the gene expression profiles up to 96 h a.d.

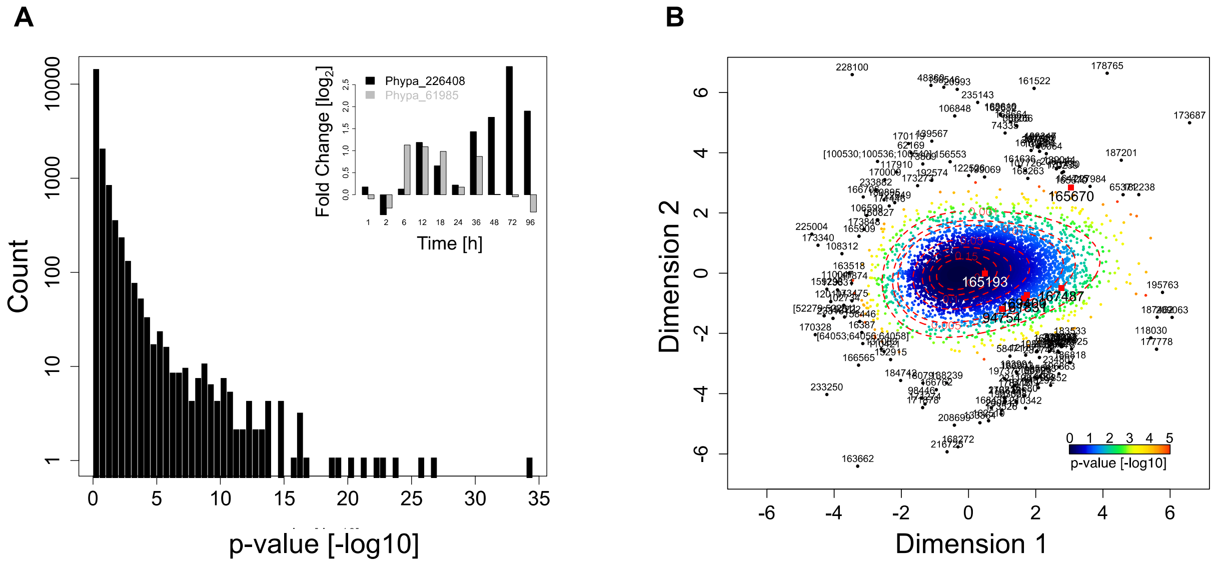

Supplement: Figure S2 — p-value histogram of transcriptome response and multi-dimensional scaling of the gene expression profiles up to 96 h a.d. A, p-value histogram for dynamic transcriptome response scores evaluated from the MDS analysis and fitting with a bivariate skew normal distribution, resulting in a long-tailed distribution. The barplot insert shows the log2 fold change dynamics of two previously known markers for apical stem cell differentiation, the transcriptional regulator PpFIE (Phypa_61985) as well as Cyclin D;1 (Phypa_226408). B, Multi-dimensional scaling (MDS) analysis of transcriptome response to leaflet detachment using 17,128 genes [35]. Symbol colors indicate the p-value of differential regulation for the whole time course. Significantly regulated genes with a FDR-corrected q-value <0.05 are marked by black dots and are additionally labeled with the P. patens gene IDs. The positions of the five predicted TFs as well as the weakly regulated PpRSL2 (165193) are additionally indicated by red squares. The red dashed lines mark curves of equal probability density by fitting a bivariate skew normal distribution to the point distribution. (PDF) [file pone.0060494.s002.pdf]
