# Supplementary material for: Network Theory Inspired Analysis of Time-Resolved Expression Data Reveals Key Players Guiding P. patens Stem Cell Development
Source: PLoS One. 2013 Apr 18;8(4):e60494. doi: 10.1371/journal.pone.0060494 (PMC3630159; doi:10.1371/journal.pone.0060494)
Supplement: Figure S4 — Protonemal filaments occasionally emerge from leaflets of slowly dehydrated gametophores. Gametophores were slowly dehydrated over several weeks in petri dishes devoid of covering laboratory film. Under these conditions, occasional emergence of protonemal filaments from leaflets of dehydrated gametophores can be seen (arrows). (PDF) [file pone.0060494.s004.pdf]

Fig. S4. Protonemal filaments occasionally emerge from leaflets of slowly dehydrated gametophores

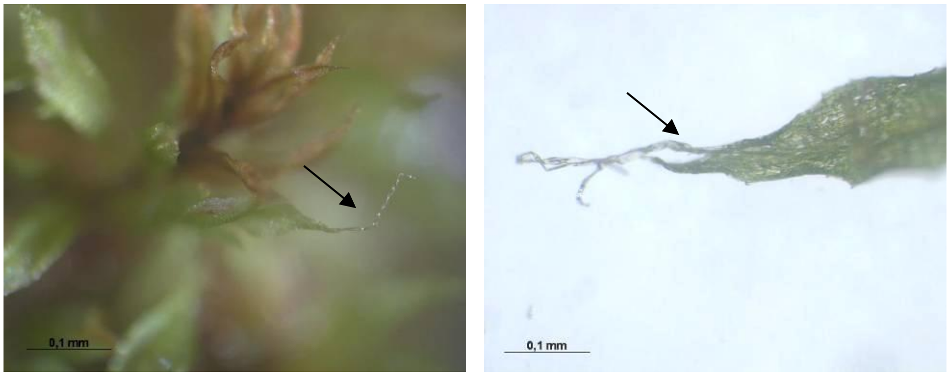

Gametophores were slowly dehydrated over several weeks in petri dishes devoid of covering laboratory film. Under these conditions, occasional emergence of protonemal filaments from leaflets of dehydrated gametophores can be seen (arrows).
