# Supplementary material for: Network Theory Inspired Analysis of Time-Resolved Expression Data Reveals Key Players Guiding P. patens Stem Cell Development
Source: PLoS One. 2013 Apr 18;8(4):e60494. doi: 10.1371/journal.pone.0060494 (PMC3630159; doi:10.1371/journal.pone.0060494)

Fig. S11. Euclidean Distance and Pearson Correlation of Gene subsets and whole transcriptome trajectory

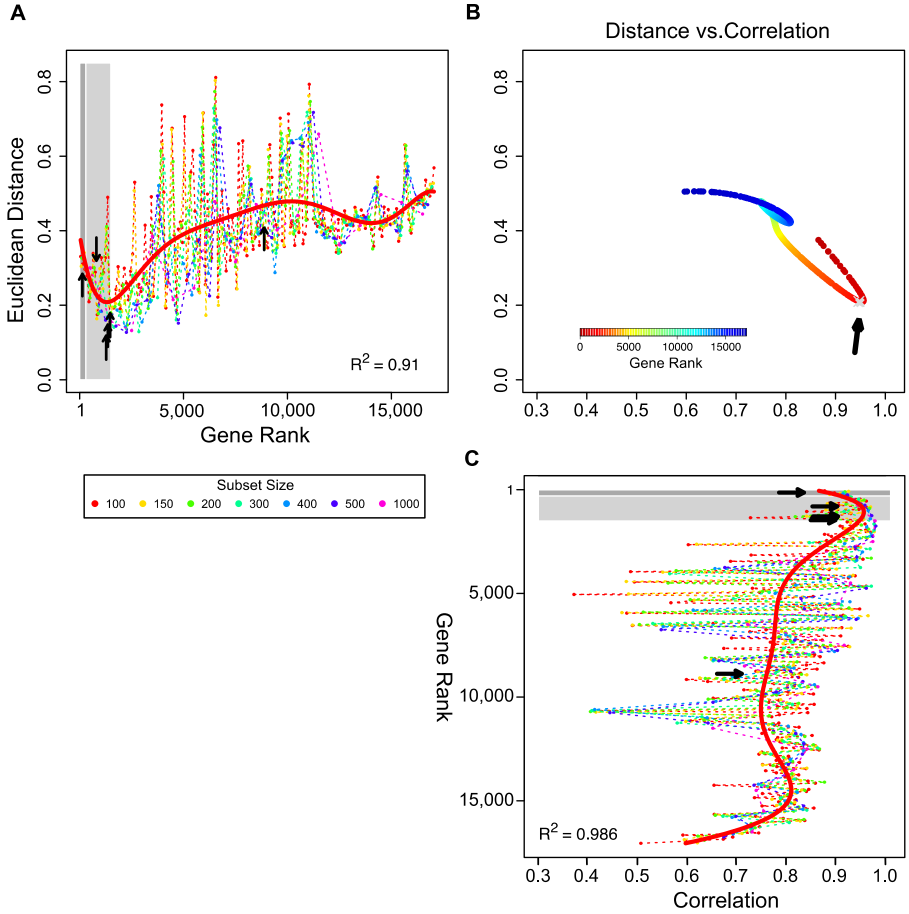

Supplement: Figure S11 — Euclidean Distance and Pearson Correlation of Gene subsets and whole transcriptome trajectory. A, Euclidean distance and C Pearson correlation of ranked gene subsets with respect to the whole genome trajectory. Both measures have their absolute maximum around gene rank 1,500. B, Projection of the Euclidean distance and Pearson correlation showing the simultaneous extremum of both measures for genes ranked between 1,000–1,500. The dark and light shaded areas in a and c mark the cutoff for significantly regulated genes from the MDS and transcriptome trajectory at rank 299 and 1,500, respectively. The cross in B marks the rank 1,500. The arrows in A and C depict the location of the five predicted TFs and of the non-involved paralog, as shown in Fig. 3. (PDF) [file pone.0060494.s011.pdf]
