# Supplementary material for: Network Theory Inspired Analysis of Time-Resolved Expression Data Reveals Key Players Guiding P. patens Stem Cell Development
Source: PLoS One. 2013 Apr 18;8(4):e60494. doi: 10.1371/journal.pone.0060494 (PMC3630159; doi:10.1371/journal.pone.0060494)
Supplement: Table S1 — Gene Ontology categories of biological processes being under-represented among the significantly regulated genes. Significantly regulated genes as detected from the MDS analysis (299 genes with a q-value <0.05). Altogether, 11,283 genes were annotated and used as background. GO categories were considered significant with a cutoff of p<0.05. (PDF) [file pone.0060494.s012.pdf]

Table S1. Gene Ontology categories of biological processes being under-represented among the significantly regulated genes.

Significantly regulated genes as detected from the MDS analysis (299 genes with a q-value < 0.05). Altogether, 11,283 genes were annotated and used as background. GO categories were considered significant with a cutoff of $p < 0.05$.

GOBPID	Term	P value
GO:0006464	protein modification process	3.07536E-24
GO:0006468	protein amino acid phosphorylation	2.90253E-22
GO:0006796	phosphate metabolic process	1.09451E-16
GO:0006350	transcription	1.51925E-15
GO:0010468	regulation of gene expression	2.76802E-15
GO:0032774	RNA biosynthetic process	8.97991E-15
GO:0006355	regulation of transcription	6.17442E-14
GO:0010556	regulation of macromolecule biosynthetic process	2.02542E-13
GO:0031326	regulation of cellular biosynthetic process	2.02542E-13
GO:0019219	regulation of nucleobase	3.96295E-13
GO:0016567	protein ubiquitination	5.03478E-09
GO:0070647	protein modification by small protein conjugation or removal	5.03478E-09
GO:0006508	proteolysis	0.0002
GO:0006470	protein amino acid dephosphorylation	0.0029
GO:0006139	nucleobase	0.0031
GO:0007018	microtubule-based movement	0.0068
GO:0044260	cellular macromolecule metabolic process	0.0076
GO:0033554	cellular response to stress	0.0089

GO:0006304	DNA modification	0.0118
GO:0065003	macromolecular complex assembly	0.0123
GO:0006281	DNA repair	0.0127
GO:0032259	methylation	0.0147
GO:0006260	DNA replication	0.0147
GO:0006952	defense response	0.0203
GO:0006306	DNA methylation	0.0203
GO:0009401	phosphoenolpyruvate-dependent sugar phosphotransferase system	0.0226
GO:0008033	tRNA processing	0.0281
GO:0006396	RNA processing	0.0319
GO:0006915	apoptosis	0.0389
GO:0006325	chromatin organization	0.0422
GO:0051336	regulation of hydrolase activity	0.0483
GO:0006352	transcription initiation	0.0483
