# Supplementary material for: Network Theory Inspired Analysis of Time-Resolved Expression Data Reveals Key Players Guiding P. patens Stem Cell Development
Source: PLoS One. 2013 Apr 18;8(4):e60494. doi: 10.1371/journal.pone.0060494 (PMC3630159; doi:10.1371/journal.pone.0060494)
Supplement: Table S2 — Oligonucleotides used for realtime PCR. (PDF) [file pone.0060494.s013.pdf]

Table S2. Oligonucleotides used for realtime PCR.

	forward primer	reverse primer
Phypa_61453	GAGGGTGGGTTCAGCACTAA	TCGTCACCTTCCTCTTTTGC
Phypa_222528	GACAGGCACAGGGTATTCCT	ATCTTCCGTCGTGTTGATCC
Phypa_165670	ACGGTTGGGTTTAAGCTGGTGTG	AGTCGTCATGCACCCTCCTAAC
Phypa_69400	ACTATCGGGGTTCCCTTCACAG	TCAGACTCTGGGGAGACCACC
Phypa_167487	ATGCAGCCTGAGACTGGGACTATG	TCCAGTTTGGTCCTTGTCATCAAC
Phypa_61985	TGGAGATGTCGTTCACATGG	GCCTAATCGGAGACTTGCAC
