# Supplementary material for: Network Theory Inspired Analysis of Time-Resolved Expression Data Reveals Key Players Guiding P. patens Stem Cell Development
Source: PLoS One. 2013 Apr 18;8(4):e60494. doi: 10.1371/journal.pone.0060494 (PMC3630159; doi:10.1371/journal.pone.0060494)
Supplement: Table S3 — qPCR validation. Log2-fold expression values relative to time point zero are shown. (PDF) [file pone.0060494.s014.pdf]

Table S3. qPCR validation.

Log2-fold expression values relative to time point zero are shown.

	Phypa_165670<br><br>bHLH TF	Phypa_167487<br><br>PpRSL1	Phypa_69400<br><br>AP2 TF	Phypa_61985<br><br>FIE
24 h	10.97	2.85	10.14	5.62
48 h	8.43	1.04	8.37	3.5
