# Supplementary material for: Network Theory Inspired Analysis of Time-Resolved Expression Data Reveals Key Players Guiding P. patens Stem Cell Development
Source: PLoS One. 2013 Apr 18;8(4):e60494. doi: 10.1371/journal.pone.0060494 (PMC3630159; doi:10.1371/journal.pone.0060494)
Supplement: Table S4 — Brachycyte formation upon ABA treatment. (PDF) [file pone.0060494.s015.pdf]

Table S4. Brachycyte formation upon ABA treatment.

	total	apical	subapical	side branch
WT	100.00%	26.03%	60.00%	13.97%
rsl1	46.33%	29.28%	50.00%	20.72%
rsl2	47.62%	43.57%	42.86%	13.57%
rsl1/2	23.93%	77.64%	8.07%	14.29%
